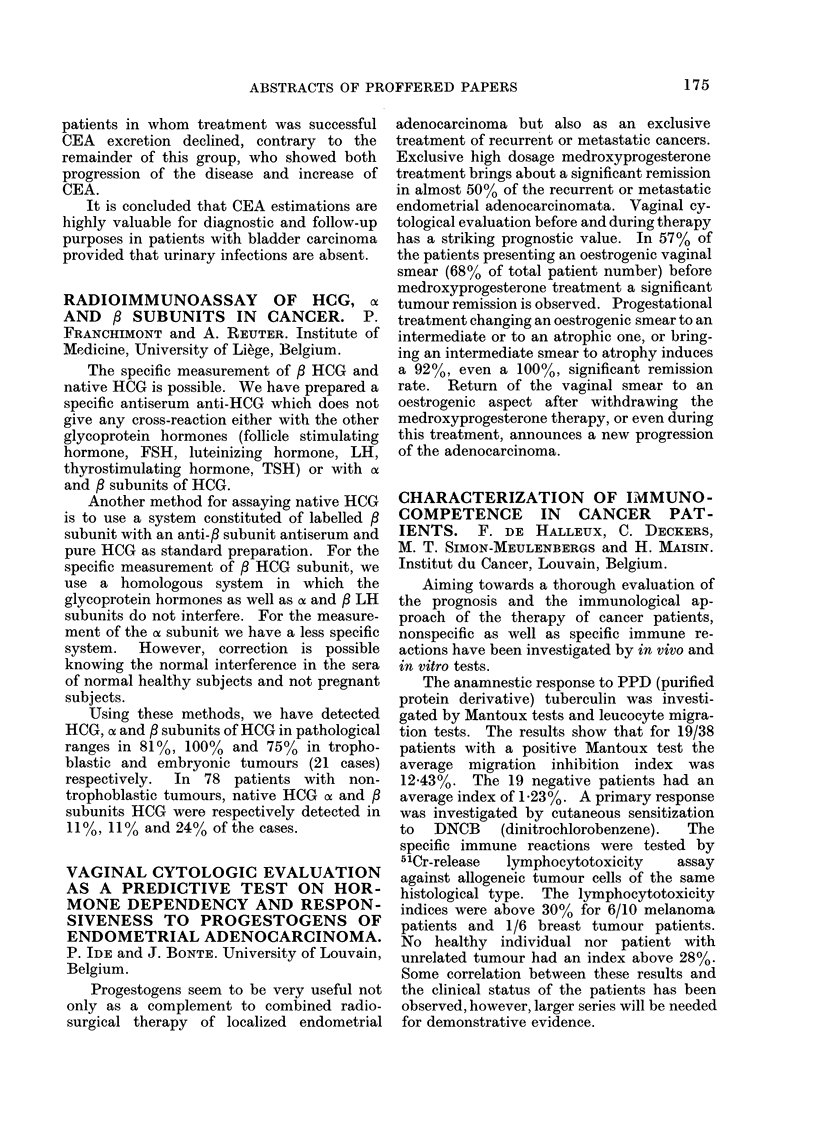# Proceedings: Characterization of immunocompetence in cancer patients.

**DOI:** 10.1038/bjc.1974.140

**Published:** 1974-08

**Authors:** F. de Halleux, C. Deckers, M. T. Simon-Meulenbergs, H. Maisin


### CHARACTERIZATION OF IMMUNOCOMPETENCE IN CANCER PATIENTS.

F. de Halleux, C. Deckers, M. T. Simon-Meulenbergs and H. Maisin. Institut du Cancer, Louvain, Belgium.

Aiming towards a thorough evaluation of the prognosis and the immunological approach of the therapy of cancer patients, nonspecific as well as specific immune reactions have been investigated by *in vivo* and *in vitro* tests.

The anamnestic response to PPD (purified protein derivative) tuberculin was investigated by Mantoux tests and leucocyte migration tests. The results show that for 19/38 patients with a positive Mantoux test the average migration inhibition index was 12·43%. The 19 negative patients had an average index of 1·23%. A primary response was investigated by cutaneous sensitization to DNCB (dinitrochlorobenzene). The specific immune reactions were tested by $^{51}$Cr-release lymphocytotoxicity assay against allogeneic tumour cells of the same histological type. The lymphocytotoxicity indices were above 30% for 6/10 melanoma patients and 1/6 breast tumour patients. No healthy individual nor patient with unrelated tumour had an index above 28%. Some correlation between these results and the clinical status of the patients has been observed, however, larger series will be needed for demonstrative evidence.